# DstarM: an R package for analyzing two-choice reaction time data with the D∗M method

**DOI:** 10.3758/s13428-019-01249-7

**Published:** 2019-05-06

**Authors:** Don van den Bergh, Francis Tuerlinckx, Stijn Verdonck

**Affiliations:** 1 Department of Psychological Methods, University of Amsterdam, Postbus 15906, 1001 NK Amsterdam, The Netherlands; 2 KU Leuven – University of Leuven, Leuven, Belgium

**Keywords:** Choice response time, Diffusion models, D^∗^M

## Abstract

The decision process in choice reaction time data is traditionally described in detail with diffusion models. However, the total reaction time is assumed to consist of the sum of a decision time (as modeled by the diffusion process) and the time devoted to nondecision processes (e.g., perceptual and motor processes). It has become standard practice to assume that the nondecision time is uniformly distributed. However, a misspecification of the nondecision time distribution introduces bias in the parameter estimates for the decision model. Recently, a new method has been proposed (called the D∗M method) that allows the estimation of the decision model parameters, while leaving the nondecision time distribution unspecified. In a second step, a nonparametric estimate of the nondecision time distribution may be retrieved. In this paper, we present an R package that estimates parameters of several diffusion models via the D∗M method. Moreover, it is shown in a series of extensive simulation studies that the parameters of the decision model and the nondecision distributions are correctly retrieved.

## Introduction

**Fig. 1:**
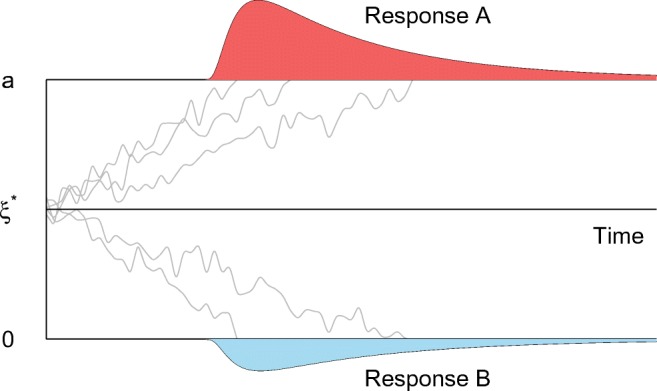
Graphical representation of a diffusion model. At the beginning of a trial, a participant’s level of information starts at *ξ*^∗^. Over time, the level of information accumulates until it reaches either boundary *a* or 0. The rate of information accumulation is the drift rate *μ*. After a decision boundary is reached, the participant makes a response. The *gray lines* represent information accumulation processes for five trials with different drift rates

Decision making is actively studied in both psychology and neuroscience. Many studies attempt to gain insight into decision processes via a combination of two-choice reaction time experiments and mathematical modeling. A number of mathematical models, collectively known as sequential sampling models, have been developed in the past decennia (see Ratcliff et al., 2016). A common assumption to all these models is that noisy evidence is accumulated (or integrated) over time to arrive at a decision. The most used and successful model from this class is the Ratcliff diffusion model (DDM; Ratcliff 1978). Following the presentation of a stimulus, a participant is accumulating information for either one of two possible responses. Once the level of accumulated information exceeds a certain boundary, the participant makes the corresponding response. The diffusion process is illustrated in Fig. 1. In the DDM, the evidence criteria are relative: If the accumulated evidence for one option goes up, the evidence decreases by the same amount for the other option. For easy stimuli, the evidence accumulates quickly to the corresponding boundary (leading to quick and accurate responses) while the opposite happens for difficult stimuli.

More formally, the DDM has a starting point *ξ*^∗^ and two boundaries *a* and 0 (corresponding to the decision criteria). The speed at which the level of information evolves is called the drift rate and is denoted *μ* (if *μ* > 0. The process tends to drift off towards the upper boundary, and vice versa). If the starting point *ξ*^∗^ lies in the middle of the boundary *a* and 0, a participant’s decision is seen as a priori unbiased for the two response options. Usually, the starting point is expressed as a proportion of the boundary (*ξ* = *ξ*^∗^/*a*) to facilitate interpretation. The information accumulation process is noisy, and the size of the noise is regulated by a standard deviation *s* (it describes how the drift rate varies within a trial). However, *s* is fixed to 1 for identification purposes.

If the same stimulus is given to the same participant repeatedly, some of the parameters will vary across these occasions; this is trial-to-trial variability. Thus, the starting point *ξ* is not a constant but can vary over trials and is therefore modeled as a uniform distribution centered at *z* with width *sz*. Similarly, the drift rate *μ* can vary over trials and is modeled as a normal distribution with mean drift rate *ν* and variance *sv*^2^. For more information on the Ratcliff model, see Ratcliff and Tuerlinckx (2002).

The DDM describes in detail what happens during the decision process. However, the total reaction time is not uniquely the result of the decision process. There are also nondecision processes playing a role and they entail everything that does not contribute to the decision making but does take up time, from the encoding of visual information to the neural representation of stimuli to eliciting a motor response (Wagenmakers, 2009). In early applications of diffusion models, the nondecision processes are modeled by a constant. Extensions of these models also estimate the variance of nondecision processes. As a consequence, these models impose a distribution on the nondecision processes. Commonly, the nondecision distribution is assumed to be uniform. However, if this assumption is violated, then bias is introduced in the parameter estimates of the decision model (Ratcliff, 2013). To circumvent specifying a distribution for the nondecision processes, a new method has been proposed, called D∗M (Verdonck & Tuerlinckx, 2016). Until now, no publicly available software application capable of doing a D∗M analysis existed. Therefore we developed the R package DstarM and provide a thorough quality check of its performance.

This paper is structured as follows. First, we provide a brief summary of the D∗M method and the Ratcliff diffusion model. Next, we provide a tutorial on how to run a D∗M analysis in R using DstarM by analyzing data from an empirical study (Wagenmakers et al., 2008). Then, we validate the performance of our implementation in DstarM via simulation studies and compare the results to those of traditional analyses. Finally, we discuss some theoretical limitations of the D∗M method and some practical issues with traditional analyses.

## The D∗M method

Assume we have data from a reaction time (RT) task with two conditions (e.g., a speed–accuracy manipulation) and two responses (e.g., correct and error). Observed RTs are (positive) random variables that can be seen as the sum of two random variables: the time spent on the decision process and the residual or nondecision time. At the level of densities, this assumption implies that the total RT density is a convolution of the nondecision time density and the decision time density:

1 $$ \begin{array}{@{}rcl@{}} f(t) = (m \ast r)(t) = {\int}_{0}^{\infty} m(t-x)r(x)\enspace \mathrm{d}x, \end{array} $$

where *f*(*t*) denotes the total RT probability density function (pdf), *m*(*t*) denotes the decision time pdf, *r*(*t*) denotes the nondecision time pdf, and ∗ denotes the convolution operator. All pdfs are a function of time *t* but we omit this in further equations for simplicity. Generally, in choice RT experiments, there are two densities for a given condition *c* (*c* = 1,…,*C*): *f*_1*c*_ for the correct and *f*_0*c*_ for the error response. Both are degenerate densities, which means that they do not integrate to one but to the probability of a correct and an error response for condition *c*, respectively. A further simplification of notation is achieved by denoting a unique condition-response pair (*c*,*x*) by a single index *p* (*p* = 1,…,*P*).

Many methods exist to estimate the parameters of *f*_*p*_ (with *p* = 1,…,*P*). Most commonly, a discrepancy measure between data and model is defined and this is directly minimized as a function of the model parameters. Such a procedure requires the specification of the nondecision distribution. The most popular software packages to estimate the diffusion model assume a uniform nondecision distribution, which has two parameters: the center and the range. However, as mentioned above, the results of such an approach may depend strongly on the specific assumption.

In contrast, the D∗M method (Verdonck & Tuerlinckx, 2016) circumvents the problem of specifying a nondecision distribution via a simple identity based on the commutative property of convolutions. Considering two distinct condition-response pairs *p* and *p*^′^ for which the same but unknown nondecision time distribution can be assumed. Then it holds that:

2 $$ \begin{array}{@{}rcl@{}} f_{p} &=& m_{p} \ast r \qquad\qquad\qquad\qquad\qquad\qquad(\text{by definition}) \\ m_{p} \ast r &=& m_{p} \ast r \qquad\qquad\qquad\qquad\qquad\ \ \ (\text{follows trivially})\\ m_{p} \ast r \ast m_{p^{\prime}} &=& m_{p} \ast r \ast m_{p^{\prime}} \qquad\qquad\qquad(\text{convolution with} \ m_{p^{\prime}})\\ m_{p} \ast r \ast m_{p^{\prime}} &=& m_{p^{\prime}} \ast r \ast m_{p} \qquad\ \quad(\text{convolution is commutative})\\ f_{p} \ast m_{p^{\prime}} &=& f_{p^{\prime}} \ast m_{p} \qquad\qquad \qquad \qquad \text{(by definition)} \end{array} $$

Equation 2 is the foundation of the D∗M: An expression is obtained that only depends on the total RT density pairs *p* and *p*^′^ and the decision densities for these pairs but not on the nondecision time distribution. When we replace *f*_*p*_ and $f_{p^{\prime }}$ by their observed counterparts ($\hat {f}_{p}$ and $\hat {f}_{p^{\prime }}$, respectively), the identity will not hold anymore. However, the parameters of the decision pdf can then be estimated by minimizing the discrepancy between the left- and right-hand side (simultaneously for multiple of such pairs):

$$ \begin{array}{@{}rcl@{}} \hat{f}_{p} \ast m_{p^{\prime}} & \approx \hat{f}_{p^{\prime}}\ast m_{p} \end{array} $$

where $\hat {f}_{p}$ is the observed RT distribution for condition-response pair *p*.

As a discrepancy, we choose to use the following Chi-square discrepancy:

3 $$ \begin{array}{@{}rcl@{}} D(a, b) = {\int}_{0}^{\infty} \frac{[a(t)-b(t)]^{2}}{a(t)+b(t)}\enspace \mathrm{d}t. \end{array} $$

This difference can be summed for every unique combination of condition-response pairs which results in the objective function *T* to be minimized as a function of the parameter vector **𝜃**:

4 $$ \begin{array}{@{}rcl@{}} T(\mathbf{\theta}) = \sum\limits_{p = 2}^{P} \sum\limits_{p^{\prime} = 1}^{p-1} D(\hat{f}_{p} \ast m_{p^{\prime}}, \hat{f}_{p^{\prime}} \ast m_{p}). \end{array} $$

In words, *T*(**𝜃**) is a function of the model parameters describing the decision distribution that calculates the sum of the difference between the left-hand side and right-hand side of Eq. 2 for all unique combinations of condition-response pairs.

One restriction must be imposed on the estimation procedure. The variance of the model distribution must be smaller than or equal to the variance of the data distribution. If we assume that the decision model and the nondecision model are independent, then the sum of their variances is equal to the variance of the data distributions. Equivalently, the variance of the nondecision distribution is equal to the variance of the total distribution minus the variance of the decision distribution. Since the variance of the nondecision distribution cannot be negative, this implies the restriction: the variance of the total distribution must be larger than or equal to the variance of the decision distribution. By enforcing that the variance of the nondecision distribution is non-negative, the procedures ensures that a nondecision distribution exists.

In the previous equations, it is assumed that all condition-response pairs have the same nondecision distribution. However, it may be the case that it is hypothesized that one set of condition-response pairs shares the same nondecision distribution, and a second set another nondecision distribution. To estimate parameters of conditions with different nondecision distributions, we calculate the objective function separately for every set with the same nondecision distribution and sum the outcome. In the most unrestricted estimation scenario, this implies that only reaction times for responses A and responses B within the same condition have an identical nondecision distribution, an assumption made by most other estimation software.

After obtaining the decision distributions, the nondecision distribution can be estimated by minimizing the following function:

5 $$ \begin{array}{@{}rcl@{}} D(\overline{\hat{f}}, \overline{m(\hat{\theta})} * \hat{r}). \end{array} $$

where the average data distribution $\overline {f}$ and average decision distribution $\overline {m(\hat {\theta })}$ are the sum of the data distributions and decision distributions divided by the number of condition-response pairs that go into them, respectively. Again, the sum only refers to condition-response pairs that were assumed to have the same nondecision distribution when estimating the parameters of the decision model. This procedure is akin to a deconvolution.

For traditional DDM analyses, we obtain parameter estimates by directly minimizing the Chi-square difference between the observed data distribution and the model distribution, for each condition-response pair.

6 $$ \begin{array}{@{}rcl@{}} T(\theta) = \sum\limits_{p=1}^{P} D(\hat{f}_{p}, m_{p}) \end{array} $$

This approach is equivalent to the Chi-square approach used in Ratcliff and Tuerlinckx (2002).

### Numerical procedures in DstarM

The D∗M procedure has been implemented in the R package DstarM. Before explaining how to use DstarM (see the next section), we discuss some technical details first. When dealing with data distributions (i.e., $\hat {f}$), we use a kernel-based approach (with a uniform kernel of bandwidth equal to 1) to derive them from the raw reaction times. The same kernel is subsequently used to smooth the average estimated decision distribution (to avoid bias). It is possible to change the default bandwidth of 1.^1^

Model distributions of the DDM are obtained via a numerical procedure (Voss & Voss, 2008) as implemented in the R-package rtdists (Singmann et al., 2016).

In DstarM, all minimizations are done using Differential Evolution, implemented in the R package DEoptim (Ardia et al., 2015; Mullen et al., 2011). To ensure full user customization, all arguments of Differential Evolution can be changed in DstarM and users can run the estimation in parallel. It is strongly advised to run the Differential Evolution procedure several times again (we settled at five) and then choose the analysis with the lowest objective function value (and hopefully there are several equal results). This is done to avoid potential convergence issues that may arise. For a more detailed explanation of the D∗M method, see Verdonck and Tuerlinckx (2016).

## A tutorial on DstarM with an empirical example

We provide a tutorial on DstarM by analyzing data from a lexical decision making task (Experiment 1 of Wagenmakers et al., 2008). These data are available in the rtdists package under the name speed_acc. Our main goal is to demonstrate how D∗M analyses can be carried out in R; we will not carry out a detailed comparison of our results with the ones obtained by (Wagenmakers et al., 2008); Verdonck and Tuerlinckx (2016) present three case studies with an in-depth comparison between the traditional DDM and the D∗M analyses (although not for the data we analyze here). We only analyze data from the first participant in the dataset to avoid needless computational complexity that does not contribute this tutorial. Furthermore, we carry out both a D∗M analysis and a traditional analysis to contrast these methods.

In this experiment, participants (*N* = 17) had to decide if a stimulus was a word or a nonword. Responses were manipulated by instructing the participants to respond either as fast as possible or as accurately as possible. A second manipulation was induced by presenting four different populations of stimuli: high-frequency words (HF), low-frequency words (LF), very low frequency words (VLF), and nonwords (NW). As observed in prior research, the first manipulation (speed/accuracy instructions) is believed to only influence the boundary parameter of the DDM. The second manipulation (word frequency) is intended to make trials harder, which is believed to influence the drift parameter of the DDM (Ratcliff et al., 2004).

### Analysis of the data from a single participant

After having installed and loaded the package (with the usual install.packages() and library() functions) the next step when using DstarM is to import the data. The data passed on to the functions of DstarM should have a structure like that in Table 1, where the first six observations of the empirical data set are shown. The data set should be a data frame (called dat in the remainder of this section) with three columns: rt containing reaction times, condition determining condition membership, and response determining response decision. Note that recoding a decision to upper or lower is arbitrary. Inverting this will only change the sign of the estimated drift speed change and flip the relative bias. In our analysis, we let upper represent ’word’ choices and lower represent nonword choices. Table 1 can be reproduced with the following code.

# get complete datasetdata('speed_acc', package = 'rtdists')# get the first six observations of thesecolumnsdf <- head(speed_acc[, c('rt', 'condition','response')])# convert 'word' to 'upper' and 'nonword'to 'lower'df\(response <- ifelse(df\)response == 'word','upper', 'lower')print(df)

**Table 1:** A snapshot of the first six observations for the first participant of the empirical data set to show the required structure of a data set when using DstarM

rt	Condition	Response
0.70	Speed	Lower
0.39	Speed	Lower
0.46	Speed	Lower
0.46	Speed	Upper
0.50	Speed	Lower
0.77	Speed	Lower

Ideally, a visual and numerical exploration of the data should be carried out before moving to more complicated analyses, but we skip that step for reasons of brevity (a more detailed description of the data can be found in (Wagenmakers et al., 2008) and code for preprocessing the raw data can be found in the rtdists package; see ?speed_acc). In order to prepare for the DstarM analyses, the analyst should provide a time grid and decide on the parameter restrictions that specify the model. First, we look into the time grid, which is usually an evenly spaced grid. We recommend a time grid from 0 to 5 in steps of 0.01 (i.e., a hundredths of a second or a centisecond). Using this grid as a standard could blur subtleties present in one-thousandths of a second, but it is unlikely that many studies hypothesize about effects that small let alone have the power to detect them. The code for defining the time grid is as follows:

# define a time gridtt <- seq(0, 5, .01)

Second, we need to specify the model by applying an appropriate set of restrictions over the parameters of different conditions (or indicating that no restrictions are needed). This is done by specifying the restriction matrix using integer values from 0 up to the number of uniquely estimated parameters. All parameters with the same integer value will be restricted to be equal. An example for the experiment of Wagenmakers et al., (2008) can be found in Table 2. The code to specify this restriction matrix in DstarM is as follows:

# restriction matrix with all parametersequal across conditionsrestr <- matrix(0:4, 5, 8)# release boundary and starting point acrossaccuracy conditionsrestr[c(1, 3), 5:8] <- 5:6# different drifts per word frequencyrestr[2, ] <- c(1, 7:9)

Once the time grid and the parameters restrictions are specified, the DDM parameters can be estimated via the D∗M method using the following code:

# estimate decision modelresD <- estDstarM(dat = dat, tt = tt, restr= restr)# estimate nondecision distributionresND <- estND(res = resD)# estimate total distributionresObs <- estObserved(resDecision = resD,resND = resND, data = dat)

The function estDstarM can also run a traditional DDM analysis where the nondecision distribution is modeled as a uniform distribution by adding the argument DstarM = FALSE. Both resD and resND are S3 class objects with custom print and plot methods. From resD, a vector containing the best parameter estimates of the decision model can be obtained by indexing with $Bestvals. The estimated nondecision distribution(s) can be obtained by running resND$r.hat. Both resD and resND can be indexed with $GlobalOptimizer to look up details about the Differential Evolution estimation procedure. The function estDstarM can also run a traditional DDM analysis where the nondecision distribution is modeled as a uniform distribution. The model then contains two more parameters: the mean and width of the uniform distribution. This must be incorporated in the restriction matrix. The code then looks as follows:

# adjust restriction matrixrestr <- matrix(0:6, 7, 8)restr[c(1, 4), 5:8] <- 7:8restr[2, ] <- c(1, 9:11)# estimate modelresT <- estDstarM(dat = dat, tt = tt, restr= restr, DstarM = FALSE)

Parameter estimates of both models are shown in Table 3. The differences in parameter estimates between the speed and accuracy manipulations are comparable between the two analyses. All ordinal relations between conditions with respect to *a* and *v* are the same for the traditional model, D∗M model and the original analyses done in Wagenmakers et al., (2008). A difference between the traditional model and the D∗M model is present in the variance parameters. It appears that the D∗M estimates attribute more variance to intertrial variability (*sv*) whereas the tradition model attributes this to variance in the nondecision distribution.

**Table 2:** An example of a parameter restriction matrix in DstarM

*a*	0	0	0	0	5	5	5	5
*v*	1	7	8	9	1	7	8	9
*z*	2	2	2	2	6	6	6	6
*sz*	3	3	3	3	3	3	3	3
*sv*	4	4	4	4	4	4	4	4

Columns represent different conditions of the experiment and rows represent parameters. The first four columns represent the speed condition, the last four columns represent the accuracy condition. The four columns within both speed and accuracy represent word frequency conditions. Identical values in the cells indicate that these parameters will be restricted across conditions

**Table 3:** Parameter estimates of the traditional model and the D∗M

	DstarM	Traditional
*a*_*acc*_	1.223	1.107
*a*_*spd*_	1.036	0.838
*v*_*HF*_	3.848	5.249
*v*_*LF*_	2.471	2.976
*v*_*VLF*_	1.351	1.660
*v*_*NW*_	− 2.407	− 2.555
*z*_*acc*_	0.486	0.463
*z*_*spd*_	0.443	0.464
*sz*	0.011	0.011
*sv*	0.588	4.280 ∗ 10^− 03^
*T*_*er*_	0.360	0.393
$\sigma _{T_{er}}$	5.501 ∗ 10^− 04^	4.353 ∗ 10^− 03^

The effect of the speed accuracy manipulation is similar in the parameter estimates of both models. Note that *T*_*er*_ and $\sigma _{T_{er}}$ represent the mean and variance of the nondecision distribution. Parameters of the uniform nondecision distribution obtained by the traditional model are *U*(*a* = 0.2791,*b* = 0.5077)

**Fig. 2:**
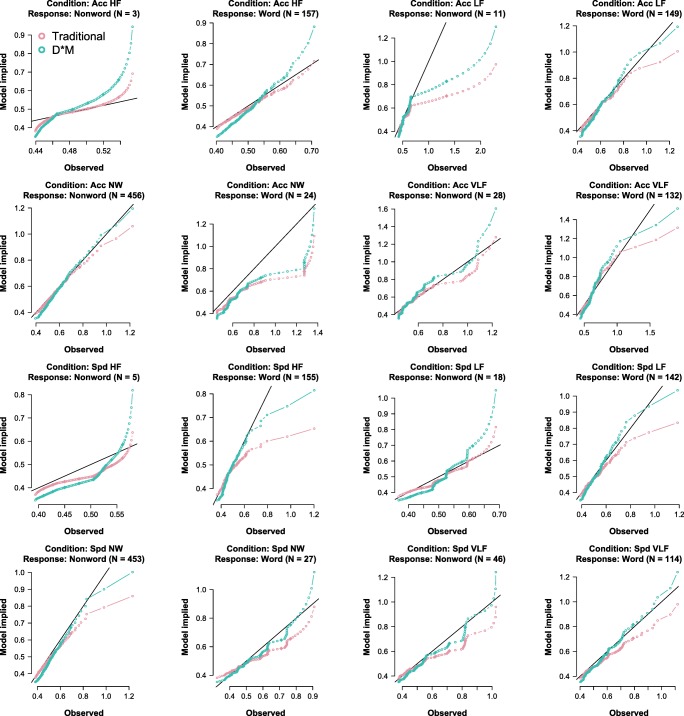
Observed quantiles (*x*-axes) vs. estimated quantiles (*y*-axes) for both the traditional model (*red*) and the D∗M model (*green*). The title of each plot indicates the condition-response pair. The abbreviations Spd, Acc, HF, LF, NW, VLF, respectively, mean speed, accuracy, high-frequency, low-frequency, very low frequency, and nonword. The sample sizes of each observed condition-response pair are shown in brackets

Next, we can compare the performance of both models. This can be done visually, as done in Fig. 2 or by comparing the *χ*^2^ goodness of fit value. For a traditional analysis, the *χ*^2^ goodness of fit is the same as the objective function. For a D∗M analysis, this has to be recalculated, which is done automatically in the function estObserved. In each case, the fit is first calculated separately for each condition-response pair. Subsequently, each individual fit is multiplied by the proportion of observations in that condition-response pair and then summed, to obtain a weighted fit measure. The resulting fit of the D∗M model was 7.638 compared to 8.030 for the traditional model.

In Fig. 2 and Table 4, it can be seen that a D∗M analysis performs somewhat better than a traditional DDM analysis. This is likely caused by the additional freedom in the shape of the nondecision distribution. The overall misfit in both analyses may be caused by the many parameter restrictions and the small sample size for many condition-response pairs. The complete script for carrying out the analyses can be found at https://osf.io/ypcqn/.

**Table 4:** Δ*χ*^2^ for all condition-response pairs

0.0001	0.1365	0.0049	0.1141
− 0.0386	0.0037	0.0149	0.2172
− 0.0010	− 0.2350	− 0.0018	− 0.1459
− 0.2241	− 0.0253	− 0.0144	− 0.1975

Each value of the 4 −×− 4 table below corresponds to the fit of one of the plots of Fig. 2. Δ*χ*^2^ is defined as $\chi ^{2}_{D*M} - \chi ^{2}_{Traditional}$. Since a lower Chi-square difference indicates better fit, negative values for the Δ*χ*^2^ indicate that the D∗M model fits better. Note that *χ*^2^ values are weighted by sample size and therefore they cannot be compared across condition-response pairs

To summarize, a D*M analysis can be carried out as follows. Before the analysis, one could get an overview of the data using rtDescriptives. This returns the observed proportions of each condition-response pair and plots density estimates for each condition-response pair.

Next, to execute the analysis, the following functions are called in order. First estDstarM, to estimate the decision model, then estND, to estimate the nondecision model, and finally estObserved, to combine the decision and nondecision model and to obtain the model implied distribution. In principle, if a researcher is interested only in the decision model, then there is no need to estimate the nondecision model. However, this means that model fit cannot be examined (e.g., Table 4 and Fig. 2 can not be obtained).

After running the analyses, a number of convenience functions allow a user to inspect the results. For instance, the call plotObserved can be used to quickly mimic Fig. 2. This function either produces QQ-plots or histograms of the data overlayed with the model-implied density. To obtain Chi-square goodness-of-fit measures (e.g., to create Table 4), the function chisqFit can be used. This returns a list containing the goodness of fit for each condition response pair (weighted by the number of observations) and the sum of the fit.

## Simulation study

### Setup of the simulation study

To validate our software tool, an extensive recovery simulation study was carried out. In the recovery study, we simulated 600 data sets, each consisting of two experimental conditions. Between these conditions, the decision model parameter values and the nondecision distributions could vary.

**Fig. 3:**
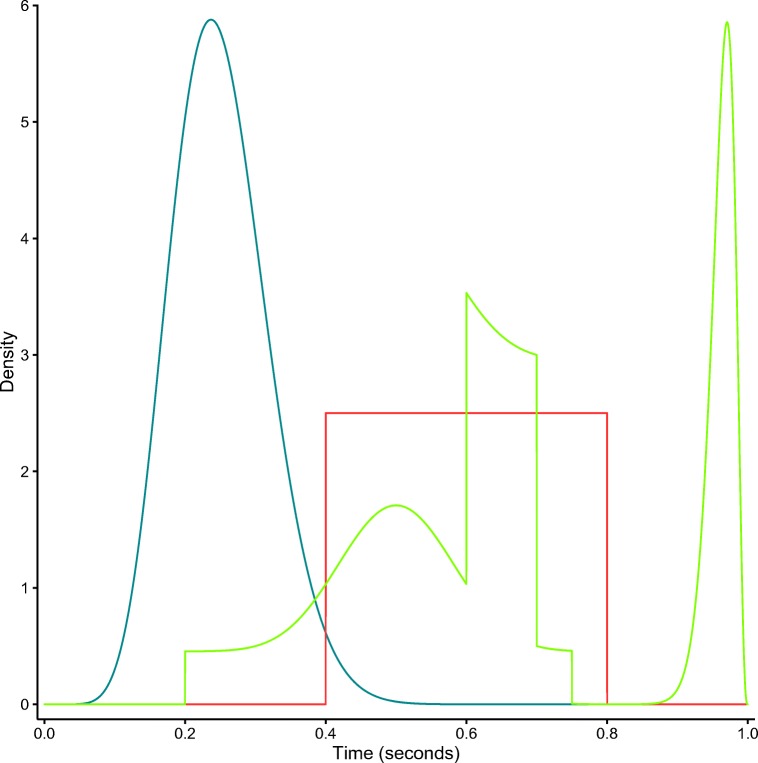
Plot of nondecision distributions used to simulate data in the recovery study. The *blue line* represents a Beta(*α* = 10,*β* = 30) distribution. The *red line* represents *U*(*a* = .4,*b* = .8) distribution. The *green line* represents a multimodal distribution obtained from the following mixture: $\frac {1}{4}\text {Beta}(\alpha = 20, \beta = 20) + \frac {1}{4}\text {Beta}(\alpha = 100, \beta = 4) + \frac {1}{4}U(a = 0.6, b = 0.7) + \frac {1}{4}U(a = 0.2, b = 0.75)]$. Here; Beta and *U* refer to the beta distribution and uniform distribution, respectively

Our data simulation procedure worked as follows. First, we created 100 sets of parameter values by drawing each parameter from an appropriate uniform distributions on the parameter space (see Table 5 for the lower and upper bounds). This resulted in 100 different parameter sets that varied widely. Next, we randomly selected a manipulation (including no manipulations) in parameters *a*, *v*, and/or *z* (e.g., if only parameter *a* was selected to be affected by manipulation, then a new *a* was drawn while the other parameters were kept constant). Then we selected for each condition one of three nondecision distributions (uniform, skewed, multimodal) at random (see Fig. 3 for details). This resulted in 100 unique sets of parameter configurations. For each of these configurations, we simulated six data sets with 100, 250, 2500, 10,000, 250,000 and an infinite number^2^ of observations per condition. All simulated datasets and the code to generate them can be found at https://osf.io/ypcqn/.

**Table 5:** Parameter ranges of the uniform distribution used to draw parameter values in the recovery study

Parameter	Lower bound	Upper bound
*a*_1_	0.5	2.0
*v*_1_	$-5.0$	5.0
*z*_1_	0.2	0.8
*a*_2_	0.5	2.0
*v*_2_	$-5.0$	5.0
*z*_2_	0.2	0.8
*sz*	0.1	0.9
*sv*	0.1	5.0

The data were analyzed with the traditional DDM (assuming a uniform nondecision distribution) and with D∗M, both using the DstarM package. As mentioned above, we analyzed every data set five times with DstarM (both for DDM and D∗M) and selected the analyses with the lowest objective function value to use in the results. This was done to avoid potential convergence issues in the Differential Evolution algorithm. For the D∗M analyses, the nondecision distributions were estimated in a next step using the best model parameters from the previous step.

### Results

This section consists of two parts. The first part reports the parameter estimates for the decision distributions. The second part shows the retrieval of the nondecision distributions.

#### Parameter estimates: Correlations with the true values and biases

Results of the simulation study are shown separately for the traditional (i.e., standard DDM with uniform nondecision distribution) and D∗M analyses in Tables 6 and 7, respectively. The left subtables contain correlations between the true and estimated parameters; the right subtables contain mean absolute relative differences between estimated and true parameters (i.e., $100 \cdot \frac {1}{N} {\sum }_{i = 1}^{N} |\frac {\hat {\theta }_{i} - \theta _{i}}{\theta _{i}} |$).

**Table 6:** Tables containing information on the recovery of the parameter values from the traditional DDM analyses)

Beta distribution												
Correlations							Relative difference (%)					
	.1	.25	2.5	10	250	$\infty $	.1	.25	2.5	10	250	$\infty $
*a*	.49	.70	.94	.98	.99	.99	30.9	22.3	12.0	7.41	4.45	3.95
*v*	.92	.94	.97	.98	.99	.99	86.5	38.4	28.9	21.2	16.5	14.3
*z*	.83	.88	.96	.97	.97	.97	24.1	15.5	9.33	8.45	8.80	7.18
*sz*	.09	$-.02$	.48	.57	.39	.43	114	117	55.9	53.6	51.7	41.6
*sv*	.48	.23	.73	.82	.84	.91	89.0	92.6	66.2	51.0	33.0	28.2
Uniform distribution												
	.1	.25	2.5	10	250	$\infty $	.1	.25	2.5	10	250	$\infty $
*a*	.52	.54	.93	.98	1.0	1.0	29.0	27.6	12.5	7.04	2.12	1.63
*v*	.87	.91	.97	.99	1.0	1.0	301	69.1	30.1	22.5	10.7	8.44
*z*	.68	.89	.98	.99	1.0	1.0	38.7	22.2	6.32	4.17	2.46	2.18
*sz*	$-.18$	$-.23$	.36	.52	.54	.61	155	166	57.6	58.0	47.4	43.8
*sv*	.43	.34	.89	.94	.94	.95	91.7	90.2	63.3	41.8	18.3	14.7
Multimodal distribution												
	.1	.25	2.5	10	250	$\infty $	.1	.25	2.5	10	250	$\infty $
*a*	.49	.52	.87	.97	.99	.99	33.9	29.4	15.5	9.21	5.46	4.89
*v*	.84	.91	.94	.97	.98	.98	459	354	87.1	41.7	34.5	36.8
*z*	.71	.89	.94	.96	.96	.97	50.9	23.3	13.9	12.4	11.8	9.53
*sz*	$-.17$	$-.12$	$-.00$	.08	.02	.05	143	173	67.2	73.3	72.4	67.4
*sv*	.44	.34	.74	.77	.78	.85	102	88.7	74.5	59.2	39.8	32.6

The left table shows correlations between estimated and true parameter values; the right table shows the mean of the absolute relative differences between estimated and true parameter values. The column headings contain the sample sizes divided by 1000 (so, the first column refers to a sample size of $0.1 \times 1000 = 100$

**Table 7:** Tables containing information on the recovery of the parameter values from D∗M analyses

Beta distribution												
Correlations							Relative difference (%)					
	.1	.25	2.5	10	250	$\infty $	.1	.25	2.5	10	250	$\infty $
*a*	.80	.94	.97	.98	.99	1.0	16.5	9.79	6.01	4.50	2.38	.005
*v*	.91	.94	.97	.97	.97	1.0	46.1	38.6	21.3	20.9	15.3	.045
*z*	.86	.92	.97	.98	.97	1.0	18.9	13.3	7.05	5.41	3.10	.051
*sz*	.16	.34	.44	.61	.84	1.0	92.4	80.1	69.2	59.9	30.1	2.14
*sv*	.39	.47	.72	.75	.68	1.0	95.2	69.9	44.2	60.4	63.2	.063
Uniform distribution												
	.1	.25	2.5	10	250	$\infty $	.1	.25	2.5	10	250	$\infty $
*a*	.78	.87	.95	.95	.98	.99	17.3	15.0	9.61	7.60	3.12	.625
*v*	.87	.89	.95	.96	.99	1.0	57.8	68.9	30.1	26.3	12.7	2.12
*z*	.84	.90	.95	.97	.99	1.0	17.5	13.7	8.76	6.18	3.27	.325
*sz*	.17	.16	.24	.35	.71	.98	89.4	102	95.2	86.2	53.0	2.14
*sv*	.26	.43	.59	.69	.86	.93	70.9	73.1	54.3	49.7	29.6	14.1
Multimodal distribution												
	.1	.25	2.5	10	250	$\infty $	.1	.25	2.5	10	250	$\infty $
*a*	.75	.82	.94	.95	.98	.99	22.6	19.2	11.6	8.56	3.06	.528
*v*	.84	.88	.92	.95	.96	1.0	74.4	53.5	40.7	40.5	23.6	1.66
*z*	.83	.78	.95	.97	.98	1.0	23.6	21.3	12.8	8.68	4.60	.263
*sz*	−.02	.28	.37	.48	.78	.98	124	103	90.7	78.3	45.8	3.47
*sv*	.25	.34	.59	.56	.66	.95	104	82.0	64.6	71.5	70.7	11.9

The left table shows correlations between estimated and true parameter values; the right table shows the mean of the absolute relative differences between estimated and true parameter values. The column headings contain the sample sizes divided by 1000 (so, the first column refers to a sample size of $0.1 \times 1000 = 100$

Evaluating Table 6, it can be seen that in general for sufficiently large sample sizes, the parameters estimated using the standard DDM analysis correlate well with their true counterparts. There are, however, important qualifications to make. First, the drift rate and boundary separation have in general higher correlations, even for small samples. Second, and to be expected, correlations are largest for the condition of a uniform nondecision distribution. Third, the multimodal nondecision distribution lowers the correlations, specifically for the trial-to-trial variability parameters.

Relative biases in Table 6 are in general quite considerable for the DDM analyses and they do not disappear completely with a large number of observations. The drift rates show more bias than boundary separation or starting point. The trial-to-trial variabilities show most bias. It may appear surprising that results from traditional analyses on datasets with uniform nondecision distributions are not unbiased as the sample size grows to infinity. This bias appears because each table describes all datasets for which at least one of the nondecision distributions used has the mentioned shape. Since parameters are restricted across conditions, this biases the results. Appendix A contains tables with parameter estimates split up per unique combination of nondecision distributions. It can be seen that the bias in parameter estimates of the traditional model for datasets with a uniform distribution disappears for larger sample sizes.

From Table 7, it can be seen that the D∗M analyses properly retrieve parameters regardless of the nondecision distribution and this is shown in relatively large correlations even for small samples (with the exception of the trial-to-trial variability parameters). The relative biases for the D∗M analyses are much smaller than for the traditional DDM analyses and they quickly become small with increasing sample size (again with the exception of the variabilities). It is striking that D∗M analyses perform also better than traditional analyses with lower sample sizes, even when the nondecision distribution is indeed uniform. We refer to Appendix B for additional scatterplots of estimated and true parameter values.

#### The estimated nondecision distribution

**Fig. 4:**
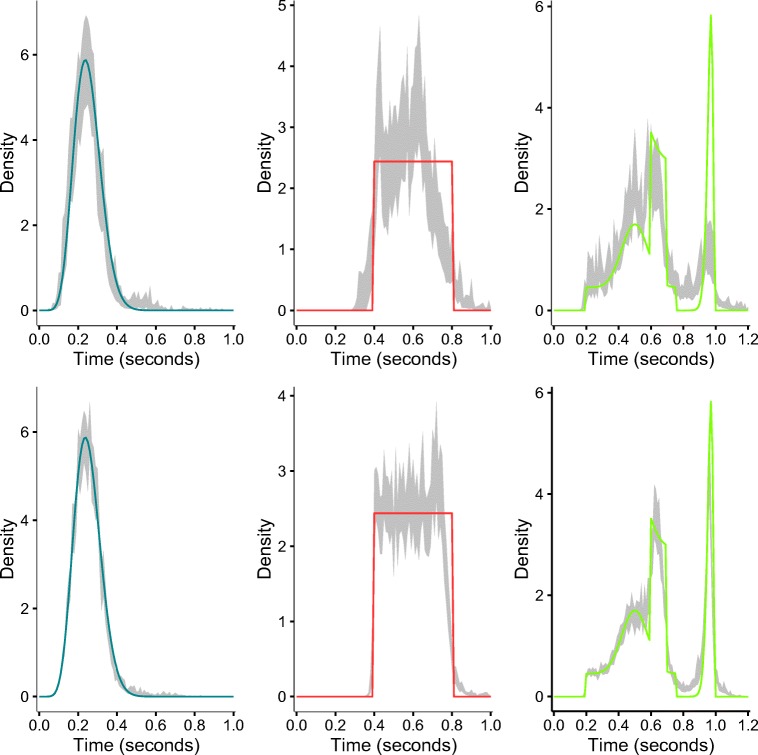
Plot of nondecision distributions estimated from data sets with 100 observations (*top three graphs*) and with 250,000 observations per condition (*bottom three graphs*). The *black line* represents the mean of the estimates and the *gray area* represents a 95% confidence interval. Confidence intervals were constructed via bootstrapping. The true distributions are plotted on top of the estimates

Figure 4 shows the (average) retrieved nondecision distributions for two sample sizes 100 and 250,000. From this plot, it can be concluded that the general shape of the nondecision distribution can be retrieved on average, even for 100 observations. Recovery is much better for 250,000 observations. Of course, it should also be remarked that a proper retrieval of the nondecision distribution depends foremost on proper estimation of the decision distribution. The smooth right-skewed beta distribution is most easy to estimate, while the procedure has most difficulties with the discontinuities and sharp corners of the two other nondecision distributions. Appendix C contains these plots for data sets with sample sizes not shown here.

**Fig. 5:**
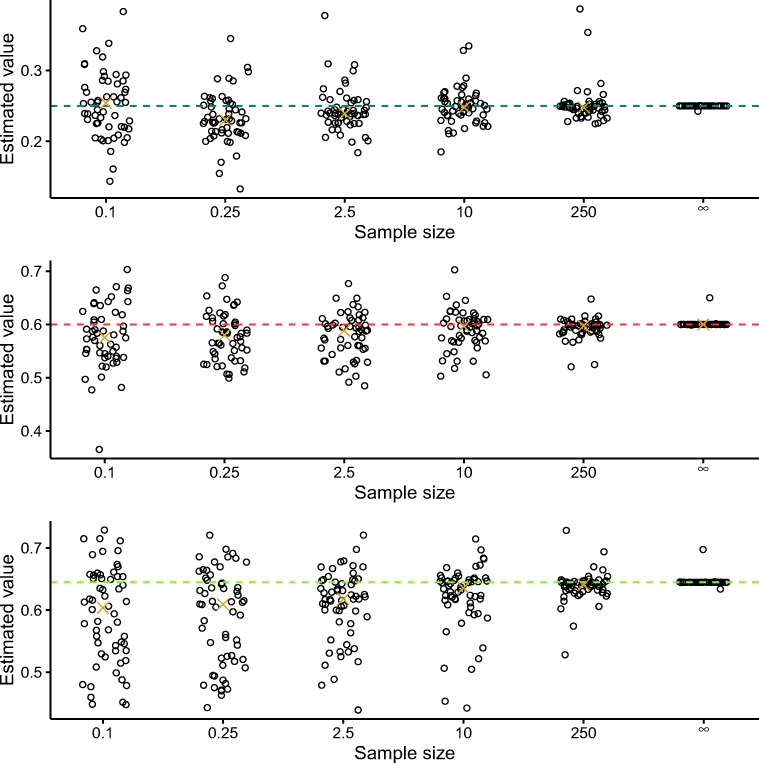
Estimated means of the nondecision distributions versus true means. The *x*-axis represents the sample sizes divided by 1000. The *gold crosses* represent the medians of the estimates and the *colored dashed lines* represent the true values. Jitter was added to the x-coordinates to improve readability

**Fig. 6:**
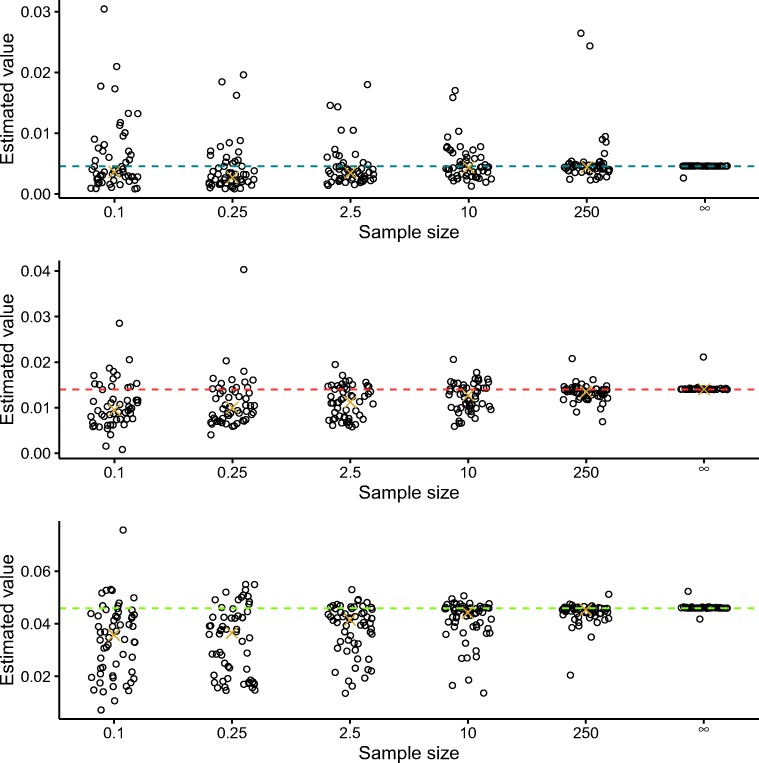
Estimated variances of the nondecision distributions versus true variance. The *x*-axis represents the sample sizes divided by 1000. The *gold crosses* represent the medians of the estimates and the *colored dashed lines* represent the true values. Jitter was added to the x-coordinates to improve readability

In Figs. 5 and 6, we show the implied estimates of the mean and variance of the nondecision distributions versus their true mean and variance. It can be seen that increasing the sample size leads to a convergence of the estimated mean and variance to their true values.

## Discussion

In this paper, we have introduced the R package DstarM to estimate the diffusion model without the need to assume a specific nondecision distribution. In an extensive simulation study, we have shown the package performs as intended. In the following sections, we discuss some limitations of the D∗M method and of traditional analyses. We also provide some design advice for researchers interested in using the D∗M method. It is worthwhile to emphasize that the D∗M is not specific to DDM analyses, but could be used with any decision model (e.g., ballistic accumulator models).

### Consequences of bias in traditional analyses due to a misspecified nondecision distribution

Results from traditional analyses may correlate highly with true values but can be severely biased as well, depending on the underlying nondecision distribution. As a consequence, conditions with misspecified and different nondecision distributions can no longer be compared meaningfully. Potential differences between conditions could be negated or increased through bias introduced by misspecification of the nondecision distributions. Effectively, results from traditional analyses should not be compared across conditions with different nondecision distributions.

### Limitations of the D∗M method

It is theoretically possible that the (observed) data distributions of condition-response pair *p* and condition-response pair $p'$ are equal. D∗M builds on the difference between these (observed) distributions (see Eq. 2). Hence, in this scenario, the D∗M method will likely encounter convergence issues or return improper parameter estimates. Of course, these situations are rare and imply that in Eq. 2*f*_*p*_ equals $f_{p'}$ with as a consequence that the models $m_{p}$ and $m_{p'}$ can have any parameters as long as their distributions are equal. This is mainly a theoretical problem and is unlikely to occur in practice.

D∗M is does not use an explicit likelihood function. Therefore, obtaining standard errors can only be done via bootstrapping. Furthermore, it is not straightforward to employ the method in a Bayesian paradigm.

D∗M currently introduces a large number of parameters for the nondecision distributions. It is important to realize that the additional parameters do not impact the estimation of the decision model parameter, because the decision and non-decision processes are separated.

The idea behind D∗M may be compared to the simple paired sample design in which for a number of persons two measurements are made. Let us denote an observation from person *i* in group *j* (with $j=1,2$) as $y_{ij}$. To account for individual differences, a person-specific parameter $\tau _{i}$ is added to the model formulation of both measurements: $y_{ij} = \alpha _{j} + \tau _{i} + \epsilon _{ij}$, where $\alpha _{j}$ is the condition effect and $\epsilon _{ij}$ (with $\epsilon _{ij} \sim N(0,\sigma ^{2}_{\epsilon })$, independently of $\tau _{i}$) is the error term. Usually we are interested in the difference between the condition effects $\alpha _{2}-\alpha _{1}$. A simple way to make inferences about this quantity is by analyzing the person-specific differences $y_{i2}-y_{i1}$. By doing so, we remove the person-specific $\tau _{i}$’s from the equation, as well as any information about their distribution.

For model selection, we advise using cross validation techniques, to avoid confusion about the status of the added non-decision parameters when calculating typical information criteria like AIC.

### Estimation of the trial-to-trial variability parameters

The variance parameters *sz* and *sv* can only reliably estimated with very high sample sizes. This issue is not inherent to the D∗M method but to the Ratcliff model since the same problem was encountered for traditional analyses. This has also been observed in reports on other estimation methods (Voss and Voss, 2007; Ratcliff & Tuerlinckx, 2002). Therefore, we discourage interpreting these parameters.

### Comparison to other estimation methods

Our implementation differs from other software used to estimate diffusion models in two aspects (aside from using a different theoretical framework). The first most notable difference is that most other software applications obtain parameter estimates by minimizing a difference between observed data statistics (e.g., the quantiles of the observed data) and their model counterparts (e.g., quantiles of the model distribution). In contrast, DstarM minimizes a Chi-square distance between (convolutions of) observed data distributions and model distributions. This Chi-square distance can roughly be interpreted as the difference between two distributions in terms of all moments.

A second difference is the optimizer used; DstarM uses a global optimization algorithm (Differential Evolution) which should make it more robust to local optima than software applications that use local search algorithms.

### Design Advice

The D∗M method performs best when there are multiple conditions that share one nondecision distribution. Obtaining conditions with quasi-identical nondecision distributions can be realized via experimental design. If an experiment consists of some conditions that only differ in stimulus difficulty then the expected effect in parameter estimates should only be present in the drift rate *v* of the Ratcliff diffusion model (Ratcliff et al., 2004). In a similar fashion, giving participants a speed or accuracy instruction is believed to only result in a change in boundary *a* of the Ratcliff diffusion model (Ratcliff & McKoon, 2008).

### Conclusions

To summarize, we have made D∗M analyses more accessible with the R package DstarM; a new method for diffusion model analyses which circumvents specifying a distribution for the nondecision processes. In simulation studies, we have shown that it performs well at retrieving parameters of a decision model and also properly estimates nondecision distributions.

## Figures and Tables

**Fig. 7:**
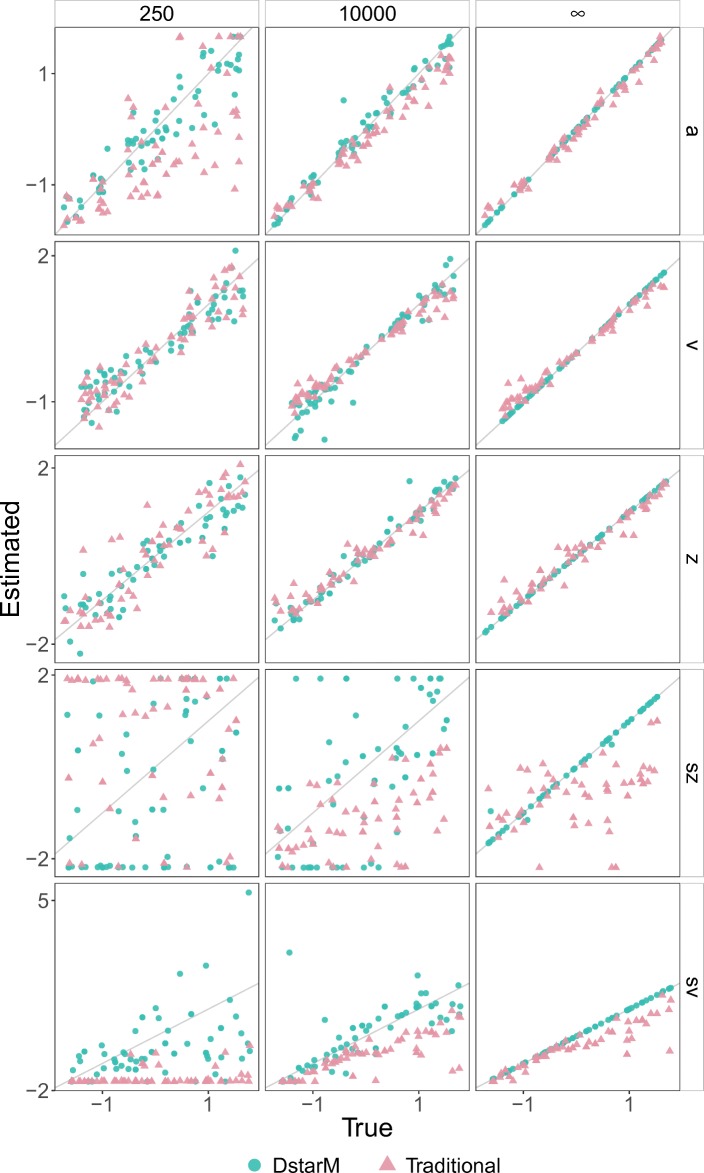
Parameter estimates of traditional analyses (*red triangles*) and DstarM analyses (*green circles*) where the nondecision time followed a beta distribution. *Columns* from left to right show results for datasets with samples sizes 250 and 10,000, and $\inf $. *Rows* indicate the parameters. Estimates were normalized with respect to the true values ($\text {est}_{\text {normalized}} = \frac {\text {est} - \mu _{\text {True}}}{\sigma _{\text {True}}}$)

**Fig. 8:**
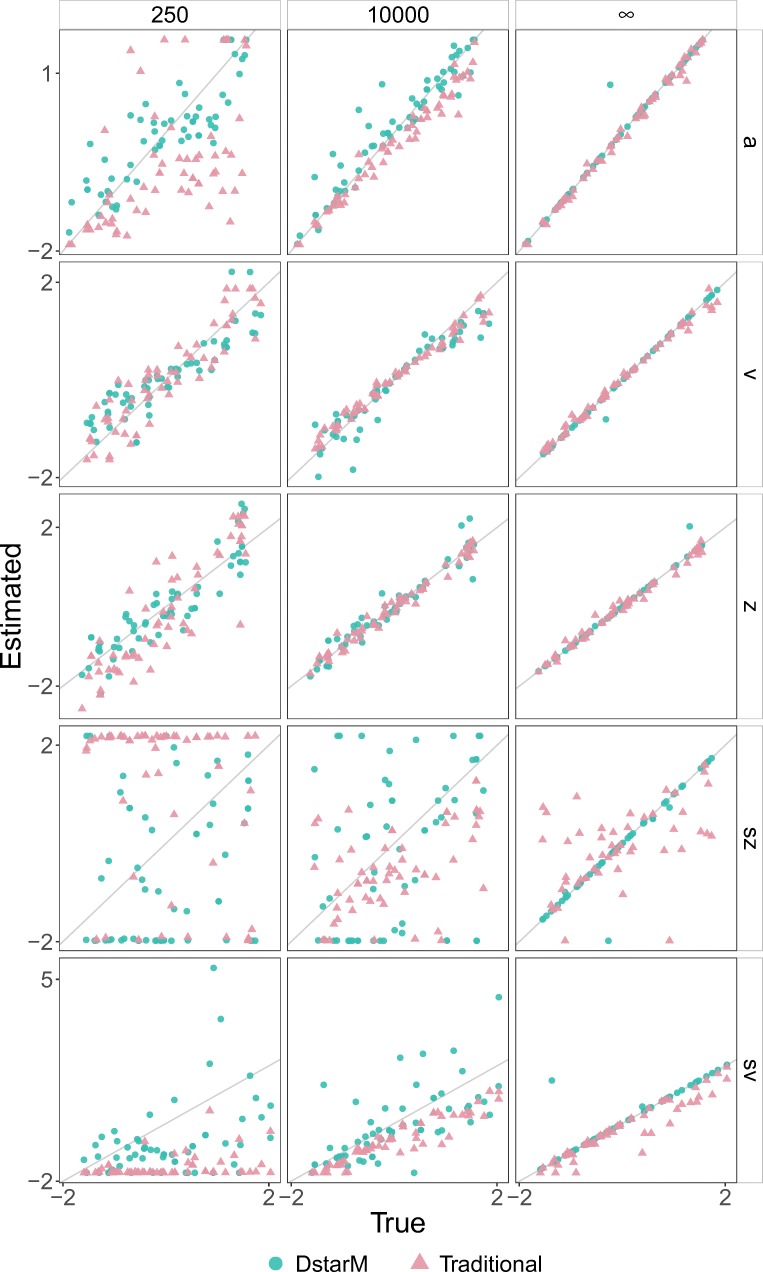
Parameter estimates of traditional analyses (*red triangles*) and DstarM analyses (*green circles*) where the nondecision time followed a uniform distribution. *Columns* from left to right show results for datasets with samples sizes 250 and 10,000, and $\inf $. *Rows* indicate the parameters. Estimates were normalized with respect to the true values ($\text {est}_{\text {normalized}} = \frac {\text {est} - \mu _{\text {True}}}{\sigma _{\text {True}}}$)

**Fig. 9:**
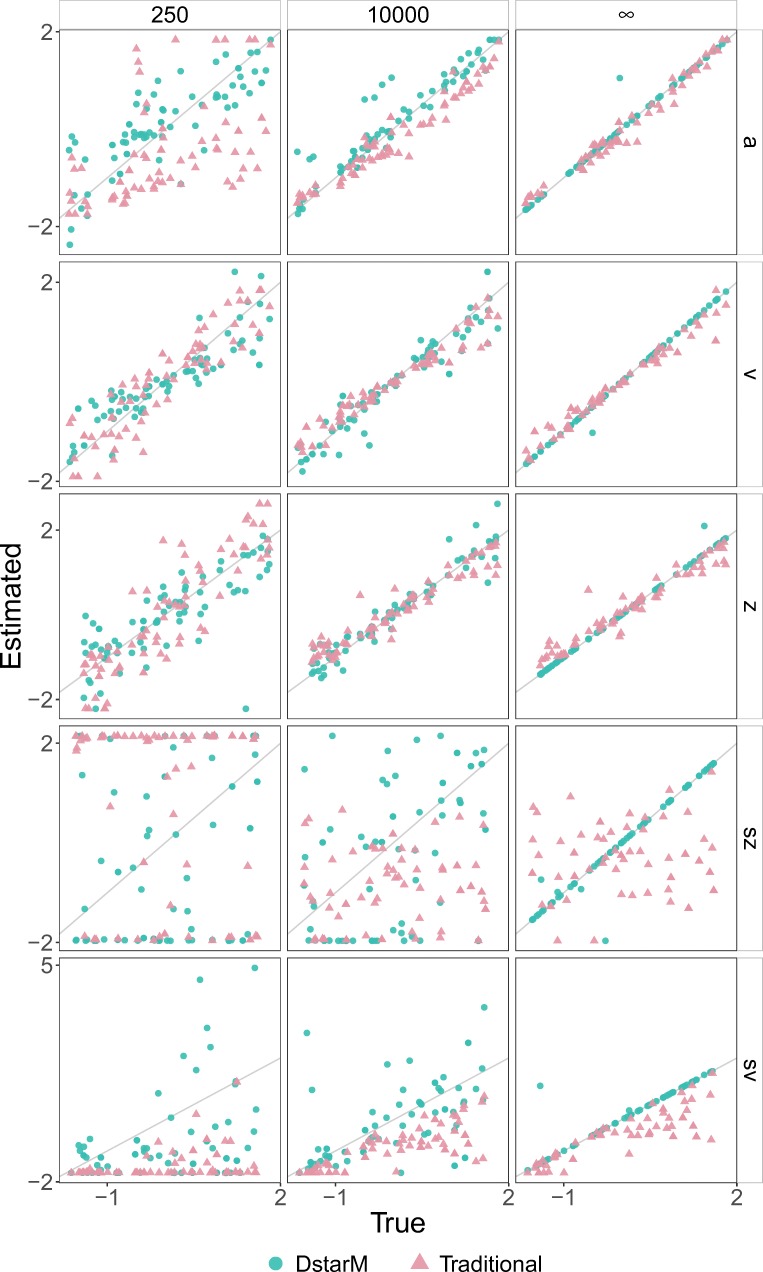
Parameter estimates of traditional analyses (*red triangles*) and DstarM analyses (*green circles*) where the nondecision time followed a bimodal distribution. *Columns* from left to right show results for datasets with samples sizes 250 and 10,000, and $\inf $. *Rows* indicate the parameters. Estimates were normalized with respect to the true values ($\text {est}_{\text {normalized}} = \frac {\text {est} - \mu _{\text {True}}}{\sigma _{\text {True}}}$)

**Fig. 10:**
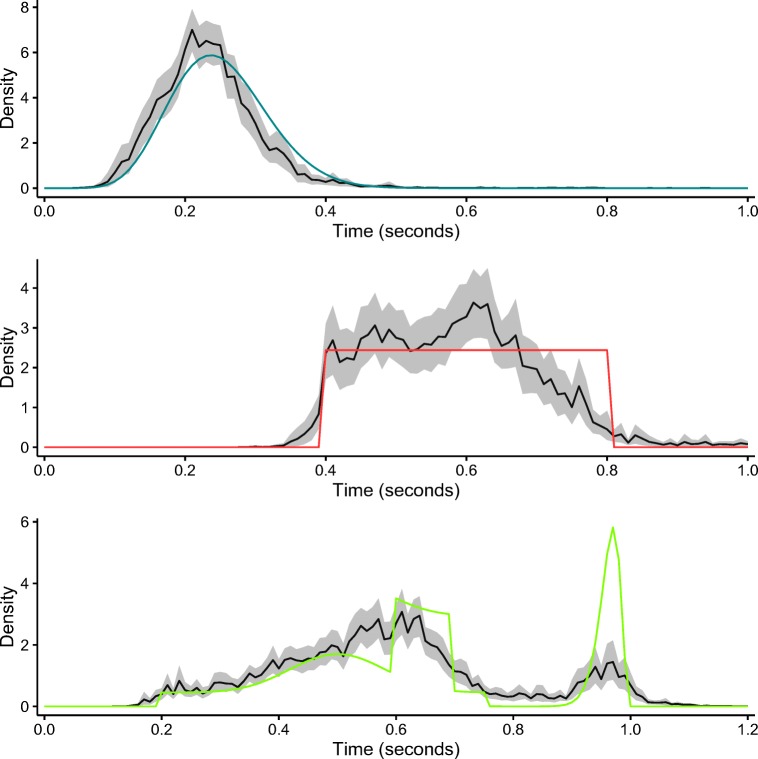
Estimated nondecision distributions for 250 observations per condition. *Colored lines* represent the true distribution

**Fig. 11:**
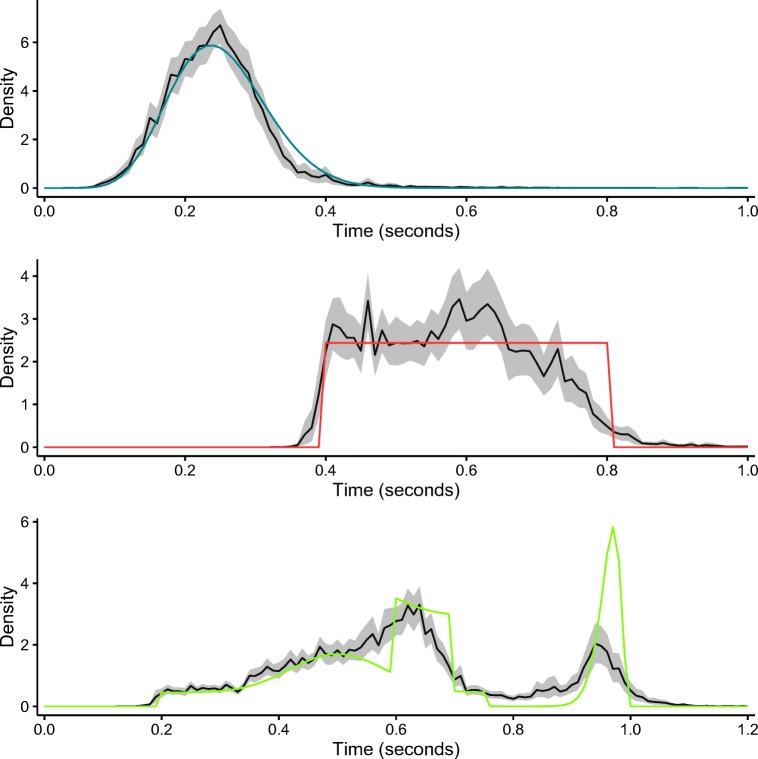
Estimated nondecision distributions for 2500 observations per condition. *Colored lines* represent the true distribution

**Fig. 12:**
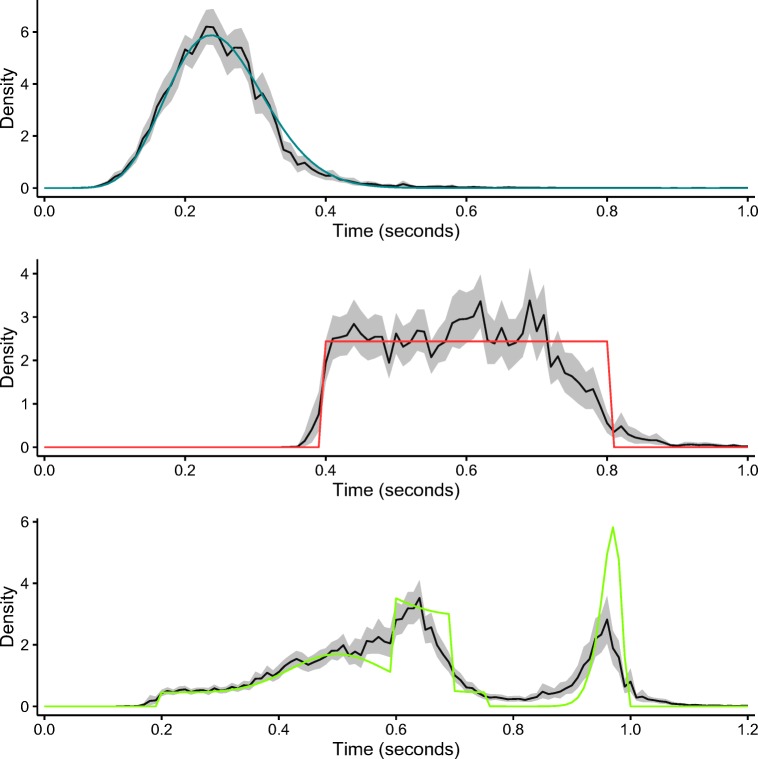
Estimated nondecision distributions for 10,000 observations per condition. *Colored lines* represent the true distribution

**Fig. 13:**
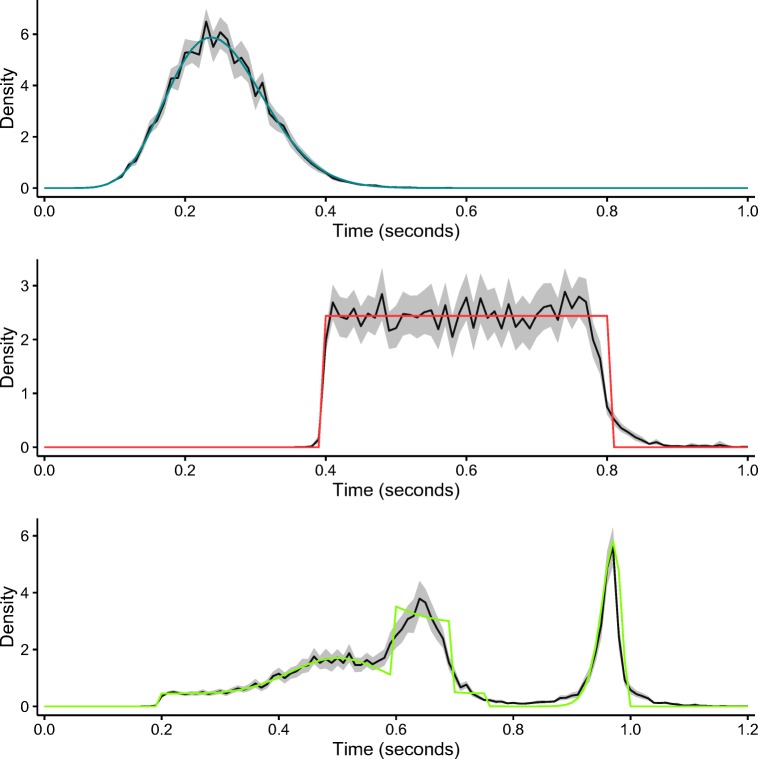
Estimated nondecision distributions for $\infty $ observations per condition. *Colored lines* represent the true distribution

## Appendix A: Parameter estimates for every unique combination of nondecision distributions

**Table 8:** Tables containing information on the recovery of the parameter values from traditional analyses

	Correlations						Relative difference (%)					
	.1	.25	2.5	10	250	$\infty $	.1	.25	2.5	10	250	$\infty $
Uniform - uniform												
*a*	.73	.76	.96	.99	1.0	1.0	24.6	20.3	9.65	5.07	.631	.000
*v*	.93	.91	.98	1.0	1.0	1.0	614	64.6	33.9	21.5	3.44	.000
*z*	.91	.94	.99	.99	1.0	1.0	20.3	21.0	5.87	4.44	.582	.000
*sz*	−.37	−.08	.71	.90	1.0	1.0	156	101	39.9	29.4	4.36	.000
*sv*	.06	.02	.89	.99	1.0	1.0	92.3	92.3	58.8	37.4	5.35	.000
Uniform – multimodal												
*a*	.45	.50	.88	.98	1.0	1.0	34.4	32.8	14.5	8.56	3.40	3.20
*v*	.85	.91	.95	.98	.99	.99	178	73.1	29.5	25.3	14.6	13.7
*z*	.65	.87	.94	.97	.98	.98	50.1	24.3	8.24	5.77	4.51	4.19
*sz*	−.11	−.38	.24	.04	.27	.43	169	248	67.8	85.9	90.5	89.5
*sv*	.47	.33	.92	.92	.92	.93	93.1	88.7	70.5	49.4	27.2	24.3
Beta - beta												
*a*	.46	.85	.95	.99	.99	.99	34.1	15.4	9.59	6.87	5.24	4.64
*v*	.96	.96	.98	.99	.99	.99	49.0	29.4	26.9	21.4	17.9	16.6
*z*	.78	.89	.95	.97	.96	.96	16.5	11.5	7.29	7.43	8.05	8.01
*sz*	.18	.22	.69	.73	.42	.56	112	88.5	57.5	54.0	61.7	45.6
*sv*	.29	−.00	.85	.90	.87	.89	96.1	94.6	69.6	56.6	38.6	34.8
Uniform - beta												
*a*	.43	.52	.93	.98	1.0	.99	32.9	28.4	13.3	7.11	2.85	2.52
*v*	.86	.91	.99	.99	1.0	1.0	142	57.0	29.6	18.4	10.5	8.92
*z*	.75	.81	.96	.98	.98	.98	32.1	22.2	6.50	4.41	4.61	4.43
*sz*	−.23	−.11	.18	.62	.45	.50	140	127	59.7	49.3	33.0	29.4
*sv*	.48	.41	.93	.96	.98	.98	89.7	90.3	58.9	37.0	18.5	15.8
Beta - multimodal												
*a*	.69	.61	.92	.98	.99	.99	25.9	27.4	14.8	8.79	5.17	4.59
*v*	.88	.91	.95	.97	.98	.99	117	53.7	36.5	27.0	22.2	18.4
*z*	.82	.93	.97	.98	.98	.98	39.8	16.6	16.1	14.5	13.6	8.88
*sz*	.23	−.16	.37	.24	.35	.19	88.9	143	49.7	57.4	58.3	50.6
*sv*	.56	.15	.52	.68	.73	.88	79.1	92.3	69.6	58.6	41.1	33.9
Multimodal - multimodal												
*a*	.52	.57	.87	.97	.98	.99	33.6	26.0	15.4	9.62	6.50	5.61
*v*	.85	.93	.94	.96	.98	.97	915	762	160	63.2	58.1	71.4
*z*	.66	.93	.94	.95	.95	.96	54.9	24.0	14.6	14.3	14.5	12.9
*sz*	−.41	.15	−.22	.21	−.09	−.10	159	135	78.4	74.2	67.9	59.9
*sv*	.39	.45	.82	.81	.81	.85	123	86.2	80.9	67.2	48.6	39.3

The table is identical to Tables 6 and 7, except that subtables are now shown for every unique combination of nondecision distributions

**Table 8:** Tables containing information on the recovery of the parameter values from D∗M analyses

	Correlations						Relative difference (%)					
	.1	.25	2.5	10	250	$\infty $	.1	.25	2.5	10	250	$\infty $
Uniform - uniform												
*a*	.88	.90	.98	1.0	1.0	1.0	18.0	14.4	7.18	2.94	1.04	.002
*v*	.85	.85	.99	1.0	1.0	1.0	75.3	105	21.2	18.7	7.59	.138
*z*	.90	.91	.98	.99	1.0	1.0	19.5	17.5	9.85	5.59	3.77	.003
*sz*	−.08	.61	.68	.25	.70	1.0	92.4	66.6	55.1	63.4	33.9	.462
*sv*	.35	.76	.81	.91	.99	1.0	56.1	72.7	29.2	26.5	7.71	.113
Uniform - multimodal												
*a*	.76	.81	.93	.92	.97	.98	23.2	22.6	15.3	13.4	5.37	1.62
*v*	.86	.88	.91	.94	.99	.99	51.1	59.5	42.1	35.7	18.9	5.20
*z*	.81	.87	.93	.95	.98	.99	18.5	15.0	9.91	7.91	4.03	.762
*sz*	.48	−.10	.05	.22	.66	.92	86.2	153	140	116	75.9	4.95
*sv*	.20	.50	.53	.57	.74	.84	86.2	85.9	84.5	79.1	57.2	36.7
Beta - beta												
*a*	.86	.97	.99	.99	1.0	1.0	14.6	7.44	5.16	3.81	1.44	.000
*v*	.95	.95	.99	.99	1.0	1.0	38.5	36.5	18.5	13.9	3.13	.000
*z*	.91	.91	.97	.99	1.0	1.0	13.9	12.0	5.35	4.35	.758	.000
*sz*	.42	.54	.54	.56	.97	1.0	72.9	69.3	64.4	54.5	17.0	.000
*sv*	.33	.61	.86	.92	.99	1.0	78.4	68.9	46.6	33.9	13.0	.000
Uniform - beta												
*a*	.72	.92	.96	.97	1.0	1.0	16.0	10.8	7.44	5.71	2.40	.012
*v*	.90	.95	.97	.95	.99	1.0	49.9	44.7	26.9	26.4	8.44	.031
*z*	.80	.93	.98	.98	.99	1.0	17.1	9.91	6.11	4.76	2.69	.092
*sz*	.09	.20	.31	.62	.78	1.0	90.4	74.6	76.3	70.7	42.4	.329
*sv*	.29	.29	.60	.78	.92	1.0	65.5	59.4	40.4	35.2	16.1	.081
Beta - multimodal												
*a*	.63	.85	.96	.96	.98	1.0	22.1	14.4	8.10	7.13	3.58	.006
*v*	.83	.91	.96	.97	.89	1.0	51.7	42.5	21.8	25.4	39.0	.138
*z*	.90	.94	.97	.98	.95	1.0	24.3	19.5	11.0	8.32	6.38	.066
*sz*	−.25	.17	.38	.64	.80	.98	120	99.8	67.9	55.8	34.1	7.36
*sv*	.45	.49	.75	.56	.27	1.0	148	82.3	45.0	121	178	.133
Multimodal - multimodal												
*a*	.89	.88	.97	.99	1.0	1.0	16.6	15.4	7.93	3.14	.815	.006
*v*	.86	.88	.92	.96	1.0	1.0	109	56.3	48.0	50.3	17.3	.017
*z*	.81	.62	.95	.97	1.0	1.0	27.5	26.3	16.1	8.93	3.53	.005
*sz*	−.21	.60	.57	.60	.89	1.0	156	65.8	68.0	64.0	30.4	.009
*sv*	.04	−.07	.54	.57	.97	1.0	87.6	79.0	62.3	32.4	9.66	.045

The table is identical to Tables 6 and 7, except that subtables are now shown for every unique combination of nondecision distributions

## Footnotes

1 Whenever a uniform kernel does not suffice, a custom density estimate can be used via the argument mg of estDstarM.

2 An infinite number observations means that we supply the algorithm with the true densities used to simulate data, instead of the actual data (where the algorithm then estimates a density from). Because our estimation procedure makes use of a distance between (empirical or theoretical) densities, it is possible to evaluate the performance of the procedure when the true densities are supplied.
